# Clinical features of colorectal cancer before diagnosis: a population-based case–control study

**DOI:** 10.1038/sj.bjc.6602714

**Published:** 2005-08-02

**Authors:** W Hamilton, A Round, D Sharp, T J Peters

**Affiliations:** 1 Academic Unit of Primary Health Care, Department of Community Based Medicine, University of Bristol, The Grange, 1 Woodland Road, Bristol BS8 1AU, UK; 2 East Devon Primary Care Trust, Unit 1, Exeter International Office Park, Clyst Honiton, Exeter, Devon, EX5 2HL, UK

**Keywords:** colorectal cancer, primary health care, diagnosis, referral and consultation

## Abstract

Most colorectal cancers are diagnosed after the onset of symptoms. However, the risk of colorectal cancer posed by particular symptoms is largely unknown, especially in unselected populations like primary care. This was a population-based case–control study in all 21 general practices in Exeter, Devon, UK, aiming to identify and quantify the prediagnostic features of colorectal cancer. In total, 349 patients with colorectal cancer, aged 40 years or more, and 1744 controls, matched by age, sex and general practice, were studied. The full medical record for 2 years before diagnosis was coded using the International Classification of Primary Care-2. We calculated odds ratios for variables independently associated with cancer, using multivariable conditional logistic regressions, and then calculated the positive predictive values of these variables, both individually and in combination. In total, 10 features were associated with colorectal cancer before diagnosis. The positive predictive values (95% confidence interval) of these were rectal bleeding 2.4% (1.9, 3.2); weight loss 1.2% (0.91, 1.6); abdominal pain 1.1% (0.86, 1.3); diarrhoea 0.94% (0.73, 1.1); constipation 0.42% (0.34, 0.52); abnormal rectal examination 4.0% (2.4, 7.4); abdominal tenderness 1.1% (0.77, 1.5); haemoglobin <10.0 g dl^−1^ 2.3% (1.6, 3.1); positive faecal occult bloods 7.1% (5.1, 10); blood glucose>10 mmol l^−1^ 0.78% (0.51, 1.1): all *P*<0.001. Earlier diagnosis of colorectal cancer may be possible using the predictive values for single or multiple symptoms, physical signs or test results.

Colorectal cancer is common worldwide, with over 30 000 new cases in the UK annually (Quinn *et al*, 2001). Survival from colorectal cancer in the UK is worse than in many other countries (Gatta *et al*, 2000a, 2003) One explanation for this may be delays in diagnosis (Gatta *et al*, 2000b). Such diagnostic delays may be reduced by a proposed national programme of screening, which has begun in pilot regions. This screening programme initially targeted those aged 50–70, with less than 60% of those invited actually participating (UK Colorectal Cancer Screening Pilot Group, 2004). Most colorectal cancers are diagnosed in patients over the age of 70, so it is likely that screening will only identify around a quarter of total colorectal cancers. Similar figures pertain in the USA, with screening recommended for those aged over 50 years (US Preventive Services Task Force, 2002), but less than half of the population have actually had a screening procedure (Seeff *et al*, 2002). Therefore, most colorectal cancers are diagnosed after symptoms have developed. This is likely to continue.

Presentation with symptoms is usually to primary care, yet most research on the symptoms of colorectal cancer has studied patients in secondary care (MacArthur and Smith, 1984; Kyle *et al*, 1991; Curless *et al*, 1994). The prediagnostic features in primary care (or any other unselected population) differ from those in secondary care, with different sensitivities, specificities and predictive values (Summerton, 2002). These parameters are largely unknown for the clinical features of colorectal cancer in primary care (Hamilton and Sharp, 2004), with positive predictive values only reported for rectal bleeding, 3.3 and 7% in two studies (Fijten *et al*, 1995; Wauters *et al*, 2000); and anaemia, 7.4% in one study (Logan *et al*, 2002). These features of cancer were examined individually in these studies, and the influence of additional symptoms on the risk of cancer has not been reported.

Once colorectal cancer is suspected, the diagnosis can only be established (or refuted) by examining the large bowel, usually by colonoscopy. This is a specialist procedure, requiring considerable investment in resources and training. Furthermore, it requires preparation of the bowel, and sedation, and has a small risk of complications, such as perforation (Robinson *et al*, 1999). In the UK, doctors who believe their patient may have a colorectal cancer can access investigation by referral to a 2-week clinic. This clinic promises rapid investigation of the suspected cancer. Referrals are aided by the Referral Guidelines for Suspected Cancer, which specify clinical features associated with cancer (Department of Health, 2000). However, the evidence base for these guidelines is weak (Hamilton and Sharp, 2004), and the majority of patients with cancer are diagnosed outwith the 2-week clinics (Boulton-Jones *et al*, 2003; Flashman *et al*, 2004). Furthermore, one risk of guidelines is that they may identify only typical presentations of cancer, whereas atypical presentations may represent early cancers, and hence may have the most to benefit from early diagnosis (Jones *et al*, 2001). Against this background, we sought to identify and quantify the features of colorectal cancer occurring before the diagnosis is made.

## METHODS

### Subjects

This was a population-based case–control study, involving all 21 general practices in Exeter, Devon, UK. The total population of Exeter in mid-2000 was 128 700, of whom 60 548 were aged 40 years or over. All patients aged 40 years or over with a primary colorectal cancer, diagnosed from 1998 to 2002, were identified from the cancer registry at the Royal Devon and Exeter Hospital. This register collects registrations from three main sources: direct notifications by clinicians, routine notification of all positive histology results and forwarding of patient lists from the oncology treatment centre. All histology and oncology treatment for Exeter patients is performed at the Royal Devon and Exeter Hospital. The register is subjected to internal and external validation procedures, and is believed to have identified over 95% of local cancers. Computerised searches at every practice identified any cases missing from the cancer register. Cases without positive histology were included if the records contained a specialist diagnosis of cancer based on strong clinical evidence. The date of diagnosis was taken as the date of positive histology or as given by the specialist in those without histology.

Five controls were matched to each case on sex, general practice, and age (to 1-year bands if possible, increased in 1-year multiples to a maximum of 5 years). The choice of five controls to each case was a balance between maximising power against decreasing efficiency. Controls were eligible if they were alive at the time of diagnosis of their case: this did not preclude their being dead at the time of study. Exclusion criteria for both cases and controls were: unobtainable records; no consultations in the 2 years before diagnosis; previous colorectal cancer; or residence outside Exeter at the time of diagnosis. Ineligible controls were replaced by randomly selected reserves. If an ineligible control was dead at the time of study, a reserve control, also dead, was used.

### Collection and coding of medical data

We made anonymised photocopies of the full primary care records for 2 years before diagnosis. Four research assistants, blinded to case/control status, coded all entries using the International Classification of Primary Care-2 (ICPC) (WONCA, 1998). This is the most symptom-based of the common coding systems (de Lusignan *et al*, 2001). Additional codes were created to incorporate all possible clinical features. Within a given general practice the same researcher coded both cases and controls, so that any interobserver variation in coding style would affect both cases and their matched controls equally. Ethical approval was obtained from North and East Devon local research ethics committee.

### Analysis

#### Identification of independent associations with cancer

Only variables occurring in at least 2.5% of either cases or controls were analysed. Differences between cases and controls were analysed using conditional logistic regression. Variables associated with cancer in univariable analyses, using a *P*-value of 0.1 or less, entered the multivariable analysis. This was performed in stages, first collecting similar variables together, such as those which could represent anaemia. These were then analysed to identify variables to progress to the second stage. These variables were re-grouped into symptoms, signs and investigations. Further multivariable analyses were then performed, first of the variables within these new groups, and then of all those variables ‘surviving’ the second stage. Using this approach, a final model was derived including all the variables independently associated with colorectal cancer. All discarded variables were then checked against the final model. Finally, 18 clinically plausible interactions were tested in the final model. Analyses were repeated excluding data from the last 180 days of the 730 day period studied.

#### Calculation of positive predictive values

This was possible because we had identified all cases occurring in the population. Positive predictive values for individual variables and for pairs of variables were calculated from the likelihood ratio and the observed incidence of cancer during the study (Knottnerus, 2002). As all cases had consulted in primary care, but 5.6% of initially selected controls had not, predictive values were divided by 0.944 to give the predictive value for the consulting population. Confidence intervals for these were calculated using Markov Chain Monte Carlo methods in Winbugs (Spiegelhalter *et al*, 2003). Stratified analyses by age (over and under 70 years) were performed for individual features, but these were not performed if any cell in the 2 × 2 table was below 10.

#### Sample size calculations

Sample size calculations used an estimated 350 cases. With this number, five controls per case were required to provide 90% power to identify a change in a rare variable from 5% prevalence in one group to 10% in the other, using a two-sided 5% alpha. This number had 94% power to identify a change in a common variable from 30% to 40%. Analyses were performed using Stata, version 8 (StataCorp, 2001).

## RESULTS

### Cases and controls

In total, 379 cases were identified from the cancer registry (376) and practice searches (3). A total of 30 were ineligible: 10 of these had previous colorectal cancer; five were unconfirmed cancers; three resided outside Exeter at diagnosis; and in 12 the records were unobtainable (seven had left Exeter, five had died). A total of 1744 matched controls were studied (in one elderly case only four controls were available in the maximum 5 year age band). In total, 1980 controls were originally generated, but 236 were ineligible: 22 had previous colorectal cancer; 111 (5.6%) had not consulted in the 2 years; 19 resided outside Exeter; and in 84 the records were unobtainable (77 had left Exeter, seven had died). These totals include 141 (40%) cases and 138 (7.9%) of controls who had died but whose notes were retrievable. In three cases, a clinical diagnosis of cancer was made but initial biopsies were negative. Positive histology was obtained later. For these, the date of diagnosis was changed to the date of the initial biopsy, 39–63 days earlier.

Of the 349 cases studied, 210 (60.2%) had tumours at or distal to the splenic flexure, and 126 (36.1%) proximal to it, with the remaining 13 (3.7%) in multiple or unknown sites. Duke's staging was known for 305 : 170 (48.7%) were Duke's A or B, and 135 (38.7%) Duke's C or D. Demographic and medical care details are shown in Table 1. For all consultation and code measures in Table 1 there was strong evidence of higher occurrence in cases than controls: *P*<0.001.

### Quality of coding

Interobserver variation in coding was examined by randomly selecting 188 codes. All four coders then coded the same records. The reliability coefficient was 0.83 (95% confidence interval 0.75, 0.90) (Streiner and Norman, 2003).

### Identification of independent associations with cancer

In total, 121 variables occurred in 2.5% or more of either cases or controls. From univariable conditional logistic regressions, 56 variables were considered for multivariable analyses. Selected univariable analyses are shown in Table 2. All variables in Table 2 were more common in cases: *P*-values <0.001 for all except history of diabetes, with *P*=0.01. Low haemoglobin results were split into three sub-categories regardless of sex: 12.9–12.0, 11.9–10.0 and <10.0 g dl^−1^. As well as conferring advantages of simplicity, this was appropriate given that the difference between the sexes in haemoglobin level diminishes markedly once women are postmenopausal, which will have been the case for almost all those in this study (Guralnik *et al*, 2004). In any case, the multivariable analysis investigated the interaction between haemoglobin subcategory and sex.

### Multivariable analyses

One ICPC code, ‘change in bowel habit,’ was excluded from modelling. This term implies constipation, diarrhoea or a combination of the two. However, when used in the UK, the phrase has connotations of suspected colorectal cancer over and above its literal meaning: the specific codes for constipation and diarrhoea were used instead.

The numbers of consultations with abdominal pain or diarrhoea followed a dose–response relationship with cancer, with the risk increasing up to the third consultation for abdominal pain and the fifth consultation for diarrhoea. These two variables were therefore kept as discrete numerical variables. The code for anal/rectal pain was associated with cancer in univariable analysis, but not so in any multivariable analyses that included rectal bleeding. Of the 349 cases, 327 had at least one of the variables from Table 3, leaving 22 cases who had none of the features. In contrast, only 532 of the 1744 controls had at least one.

There was an interaction (*P*=0.016) between haemoglobin subcategory and age group, whereby the association between anaemia and cancer was stronger among younger patients. To illustrate this, the likelihood ratios (95% confidence interval (CI)) for the younger age group (aged 40–69) were haemoglobin 12.9–12.0 g dl^−1^, 15 (6.8, 33), haemoglobin 11.9–10.0 g dl^−1^, 17 (10, 29) and haemoglobin <10 g dl^−1^, 13 (6.7, 27). For patients aged over 70 years the likelihood ratios were 3.1 (1.7, 5.5), 2.7 (1.8, 3.9), and 8.9 (6.4, 12), respectively. Including this interaction in the model made virtually no difference to its performance in terms of risk prediction and would have made it considerably more complex; hence, it was omitted from the model presented in Table 3.

Two antagonistic interactions were retained in the final model: abdominal pain with abdominal tenderness, and positive faecal occult bloods with a haemoglobin<10 g dl^−1^. These interactions reflect the fact that the combined effect of, for example, both positive faecal occult bloods and low haemoglobin together is less than that which would be expected by simple multiplication of the odds ratios in Table 3. There were no interactions with sex, supporting the decision to use the same haemoglobin subcategories for both sexes.

### Timing of variable occurrence and analysis excluding the last 180 days

**Figure 1:**
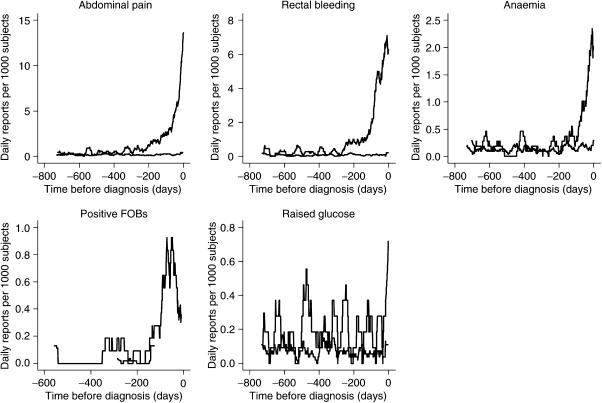
Timing of symptom presentation to primary care in cases and controls. Time 0 is the date of diagnosis in the case. Results presented as monthly moving average. Upper line=cases, lower=controls. *Y*-axis has different scales.

Multivariable analysis using data excluding the last 180 days is shown in Table 4. The timings of the five variables from Table 4, in relation to the date of diagnosis, are shown in Figure 1. These graphs compare the monthly moving average number of presentations to primary care for each variable.

### Positive predictive values for patients consulting a doctor in primary care

**Figure 2:**
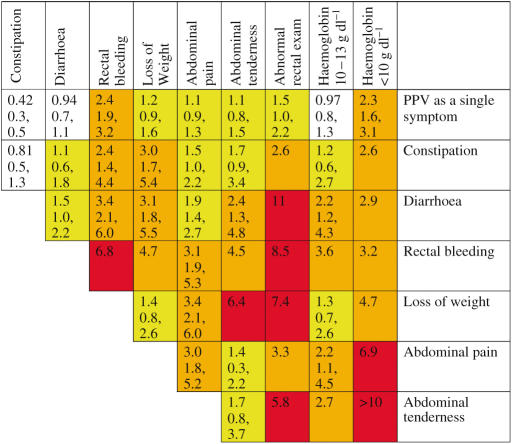
Positive predictive values for colorectal cancer for individual features, repeat presentations and for pairs of features (in the context of a background risk of 0.25%). The top row gives the positive predictive value (PPV) for an individual feature. The cells along the diagonal relate to the PPV when the same feature has been reported twice. Thus, the constipation/constipation intersect is the PPV for colorectal cancer when a patient has attended twice (or more often) with constipation. Other cells show the PPV when a patient has two different features. The top figure in each cell is the PPV. It has only been calculated when a minimum of 10 cases had the feature or combination of features. The two smaller figures are the 95% confidence intervals for the PPV. These have not been calculated when any cell in the 2 × 2 table was below 10. For haemoglobin <10 g dl^−1^ with abdominal tenderness, no controls had this pair. It was scored as a PPV of >10%. The yellow shading is when the PPV is above 1%. The amber shading is when the PPV is above 2.5%, which approximates to a risk of colorectal cancer of 10 times normal. The red shading is for PPVs above 5.0% approximating to a risk of 20 times normal.

The positive predictive values for clinical features are shown in Figure 2, both individually and together with a second variable. The variables chosen were those from the multivariable analysis in Table 3, apart from a raised glucose or a positive faecal occult blood, as these had small numbers. Haemoglobin values between 12.9 and 10.0 g dl^−1^ were merged for this analysis, as their likelihood ratios were similar.

The positive predictive values of the following features were higher for older patients: abdominal pain 0.65% aged 40–69, 2.0% aged 70 or over; diarrhoea 0.63 and 1.7%; constipation 0.2 and 1.3%; rectal bleeding 1.4 and 4.8%; and loss of weight 0.74 and 2.5%, respectively. There were too few patients aged 40–69 with the other variables for reliable analysis.

## DISCUSSION

We found 10 symptoms, signs or investigation results to be independently associated with colorectal cancer. Five of these remained associated with cancer 180 days before diagnosis. As well as identifying these features, we were able to quantify the risk they pose, both alone and in combination.

### Study strengths and weaknesses

This is the first study to examine all the prediagnostic features of colorectal cancer together. We were also able to study many more cases than in previous reports of single clinical features from primary care (Fijten *et al*, 1995; Wauters *et al*, 2000; Logan *et al*, 2002). Furthermore, as every general practice in Exeter participated in the study, we could identify all the cases occurring in a well-defined population, and studied almost all of them. The Dendrite Register is believed to be very complete: this view is supported by the fact that only three additional cases were found on practice searches. Practice recordkeeping was also good, as shown by our separate study of the quality of the notes (Hamilton *et al*, 2003). We were also able to estimate the proportion of the population consulting with their doctor, and thus could calculate positive predictive values for the consulting population. However, by only studying patients who had consulted, our results do not provide the frequency or the predictive values of symptoms in the general population. This may not matter, in that the study was stationed where the clinical problem exists: when to investigate a patient who has a problem that may represent colorectal cancer.

The first potential weakness of the study is that recording of symptoms and signs may vary between practices. This is less of an issue for test results, as these were extracted directly from the laboratory printout. It is possible that doctors record symptoms more thoroughly if they consider cancer a possibility. If this is the case, the positive predictive values in this study will have been overestimated. The converse – of more recording of symptoms when no diagnosis is apparent – is also possible, but less likely. The matched design will have partly compensated for such variations in testing and recording.

However, matching can be a weakness too. By matching, the ability to study the matched variable directly is lost. The two major factors affecting primary care consultation rates are age and sex (McCormick *et al*, 1995). These had to be matched for, and the final decision to select randomly from a 1-year age band was a careful balance between insufficient and over-matching. The third factor we matched was the general practice. This was to reduce bias from different recording methods, particularly whether the doctors used paper systems or a computer.

The large number of variables that were eligible for multivariable analysis (because of the liberal 10% threshold employed at the univariable stage) raises the possibility of false positive associations. The exhaustive, structured, approach to the multivariable analyses should have identified such influences. In the final model, there was a manageable number of variables, all with very strong evidence of associations both in terms of magnitude and statistical significance. All of the variables in the final multivariable model have been reported with colorectal cancer before. However, in observational epidemiology, residual confounding and false positives can never be entirely ruled out.

### Symptoms

The level of risk above which investigation is appropriate depends on the viewpoints of the patient, the doctor and the health care organisation. The choice of a threshold level for referral has considerable resource implications, with a lower threshold level requiring greater provision of investigative services. The percentage of colorectal cancers found in two reports of referrals to 2 week clinics was 9.4 and 16% (Boulton-Jones *et al*, 2003; Flashman *et al*, 2004). This suggests that the Referral Guidelines for Suspected Cancer – if they were used by the referring doctors – have a high specificity, but possibly at the cost of a low sensitivity. Our positive predictive values may be a better guide. We consider a risk of 2% or more justifies investigation, though many would argue for a lower threshold.

The positive predictive value of rectal bleeding was 2.4%. This is less than the 3.3 and 7% reported from two previous primary care reports, although these studied only 9 and 27 cancers, respectively (Fijten *et al*, 1995; Wauters *et al*, 2000). In patients aged under 70, the positive predictive value was 1.4%. Rectal bleeding occurred in 4.2% of controls over 2 years, a remarkably similar finding to the 2.1% of the normal population in 1 year previously reported (Fijten *et al*, 1993). This also suggests that retrospective use of primary care records, as in this study, is not associated with an important loss of data. These figures largely support current UK Referral Guidelines, which suggest investigation of patients over 60 with persistent rectal bleeding, unaccompanied by local anal symptoms (Department of Health, 2000). The ICPC code nearest in meaning to the umbrella term ‘local anal symptoms’ has a slightly different definition, that of anal/rectal pain. Thus, we were unable to study if local anal symptoms (which includes other symptoms such as itching) do indeed reduce the risk that rectal bleeding is due to cancer, although anal/rectal pain did not appear to do so.

Diarrhoea and constipation were both associated with cancer in the multivariable analyses. It is debatable whether the predictive values for diarrhoea of 0.63% in patients under 70, and 1.7% in those over 70, warrant investigation of this symptom on its own. However, the presence of any second variable increased the risk of colorectal cancer to a level when most doctors would consider investigation. Constipation is less of a risk, supporting the recommendations in the UK Referral Guidelines (Department of Health, 2000).

Abdominal pain is a difficult problem for the doctor in primary care; if anything our results compound this difficulty. It is a very common symptom, yet it is associated with colorectal cancer. Furthermore, abdominal pain and rectal bleeding are the two symptoms that retained their association with the cancer 180 days before diagnosis. Serious consideration should be given to the possibility of cancer with abdominal pain and no clear diagnosis. This would include asking about other symptoms, performing abdominal and rectal examinations, and testing of faecal occult blood and haemoglobin. Positive findings on any of these would suggest referral for investigation of possible colorectal cancer.

### Investigations

Anaemia has long been recognised as a feature of colorectal cancer. Some of the cases in this study had low haemoglobin results more than a year before eventual diagnosis. Furthermore, haemoglobin results between 12.0 and 12.9 g dl^−1^ were associated with cancer, yet some laboratories label results in this range as normal. Doctors are more likely to investigate anaemia if there are accompanying symptoms (Yates *et al*, 2004), and our results confirm that the risk of cancer with anaemia is indeed higher when symptoms are also present. However, the predictive value of haemoglobin below 10 g dl^−1^ *per se* was 2.3%. This supports recommendations that all patients with iron deficiency anaemia are investigated (Goddard *et al*, 2000).

The predictive value of 7.1% for a positive faecal occult blood test in this study is similar to the 10.9% in those investigated after a positive test in the UK screening pilot, and mandates investigation (UK Colorectal Cancer Screening Pilot Group, 2004). This suggests that any bias introduced by selective testing of patients (as suggested by the figures in the footnote to Table 2) is small. In any event, such influences would be largely accounted for by controlling for presenting signs and symptoms in the multivariable analysis. The antagonistic interaction between a positive faecal occult blood and severe anaemia probably arises from the fact that both features represent gastrointestinal bleeding. Once one of the features is present, the addition of a second one does not increase the risk as much. The association between raised glucose and cancer has been reported before in prospective studies (Trevisan *et al*, 2001; Saydah *et al*, 2003). As diabetic patients have an increased risk of colorectal cancer, consideration has to be given to the possibility when a patient presents with one of the other features (Coughlin *et al*, 2004).

## CONCLUSIONS

In patients who have been referred for investigation of possible colorectal cancer, the predictive values for symptoms are much higher than in the study reported here (Selvachandran *et al*, 2002). For example, rectal bleeding in the referred population had a positive predictive value of 5.2%, and weight loss 9.4%, compared with our 2.4 and 1.2%, respectively. Our findings come from primary care, and should be a more accurate guide for clinicians who manage unselected patient populations. The positive predictive values give an initial guide when a single feature, or pair of features, is present. The implications of combinations of symptoms can be gleaned from the multivariable analysis. Our findings can also be used to develop guidelines to select patients for rapid investigation. There may be as much – or more – benefit to be achieved from earlier diagnosis of symptomatic colorectal cancer as from screening for asymptomatic cancer. The two approaches are complementary. An important minority of colorectal cancers, or their precursors, colorectal polyps, will be identified by screening at an asymptomatic stage. Symptomatic patients may benefit from early diagnostic tools used in primary care, based on the symptoms and investigation findings in this study. A feasibility study of such a tool begins in 2005, with further research required to examine its utility.

## Figures and Tables

**Table 1:** Characteristics of colorectal cancer cases and matched controls

**Characteristic**	**Cases (*N*=349 (%))**	**Controls (*N*=1744 (%))**
*Age at diagnosis*		
<60	45 (12.9)	225 (12.9)
60–69	97 (27.8)	487 (27.9)
70–79	113 (32.4)	555 (31.8)
80+	94 (26.9)	477 (27.4)

*Sex*		
Male	177 (50.7)	885 (50.7)

	**Median (interquartile range)**	
*Number of consultations per patient*		
In the two years	15 (8–22)	10 (5–17)
Excluding last 180 days	9 (4–15)	7 (4–13)

*Number of ICPC codes per patient*		
In the two years	33 (19–50)	19 (10–33)
Excluding last 180 days	18 (7–31)	13 (7–24)

**Table 2:** Univariable analyses of selected variables

	**Number (%) with this variable present**		
**Variable**	**Cases (*n*=349)**	**Controls (*n*=1744)**	**Positive likelihood ratio (95% CI)**
*Symptoms*			
Rectal bleeding	148 (42.4)	73 (4.2)	10 (7.9, 13)
Loss of weight	94 (26.9)	92 (5.3)	5.1 (3.9, 6.6)
Abdominal pain	148 (42.4)	163 (9.4)	4.5 (3.8, 5.5)
Diarrhoea	132 (37.8)	171 (9.8)	3.9 (3.2, 4.7)
Constipation	91 (26.1)	258 (14.8)	1.8 (1.5, 2.1)

*Physical signs*			
Rectal disease on rectal examination	51 (14.6)	14 (0.80)	18 (10, 32)
Tenderness on palpation of abdomen	62 (17.7)	67 (3.8)	4.6 (3.3, 6.4)

*Investigations*			
Positive faecal occult blood^a^	31 (8.9)	5 (0.3)	31 (22, 43)
Haemoglobin 12–12.9 g dl^−1^	17 (4.9)	20 (1.2)	4.3 (2.7, 6.8)
Haemoglobin 10–11.9 g dl^−1^	38 (10.9)	49 (2.8)	3.9 (2.8, 5.2)
Haemoglobin <10 g dl^−1^	40 (11.5)	21 (1.2)	9.5 (7.1, 13)
Blood sugar >10 mmol l^−1^	25 (7.1)	39 (2.2)	3.2 (2.2, 4.7)

*Miscellaneous*			
History of diabetes	37 (10.6)	119 (6.8)	1.6 (1.2, 2.1)

a From the 79 (23%) cases and 47 (3%) controls who had been tested.

**Table 3:** Multivariable conditional logistic regression analysis of prediagnostic features of colorectal cancer

**Variable**	**Odds ratio**	**95% CI**	***P*-value**
*Symptoms*			
Rectal bleeding	15	9.0, 24	<0.001
Loss of weight	2.7	1.7, 4.6	<0.001
Number of episodes of abdominal pain^a^	2.2^b^	1.7, 2.8	<0.001
Constipation	2.0	1.2, 3.3	0.006
Number of episodes of diarrhoea	1.6^b^	1.3, 2.0	<0.001

*Signs*			
Rectal disease on rectal examination	13	4.7, 37	<0.001
Tenderness on palpation of abdomen^a^	3.6	1.7, 7.8	0.001

*Investigations*			
Positive faecal occult blood^a^	81	20, 330	<0.001
Low haemoglobin, category			<0.001
No low haemoglobin	1		
Haemoglobin 12.0–12.9 g dl^−1^	2.5	0.95, 6.8	
Haemoglobin 10.0–11.9 g dl^−1^	4.3	2.1, 9.0	
Haemoglobin <10 g dl^−1^^a^	13	6.2, 28	
Blood sugar >10 mmol l^−1^	2.0	1.3, 3.1	0.001

*Interaction terms*			
Abdominal pain with tenderness	0.56	0.38, 0.82	0.003
Positive FOBs with haemoglobin <10 g dl^−1^	0.020	0.0015, 0.27	0.003

a Variables that have interactions.

b For each consultation with this symptom.

**Table 4:** Multivariable conditional logistic regression analysis of prediagnostic features of colorectal cancer excluding the final 180 days

**Variable**	**Odds ratio**	**95% CI**	***P*-value**
*Symptoms*			
Rectal bleeding	3.0	1.9, 4.9	<0.001
Abdominal pain	2.3	1.6, 3.2	<0.001

*Investigations*			
Positive faecal occult blood	5.4	1.2, 25	0.029
Low haemoglobin, category			<0.001
No low haemoglobin	1		
Haemoglobin 12.0–12.9 g dl^−1^	2.3	1.2, 4.3	
Haemoglobin 10.0–11.9 g dl^−1^	3.0	1.8, 5.0	
Haemoglobin <10 g dl^−1^	7.1	3.7, 14	
Blood sugar >10 mmol^−1^l^−1^	1.8	1.4, 2.4	<0.001
